# Differential Activation of ERK Signaling in HPV-Related Oropharyngeal Squamous Cell Carcinoma

**DOI:** 10.3390/cancers11040584

**Published:** 2019-04-25

**Authors:** Chao Rong, Marie Muller, Christa Flechtenmacher, Dana Holzinger, Gerhard Dyckhoff, Olcay Cem Bulut, Dominik Horn, Peter Plinkert, Jochen Hess, Annette Affolter

**Affiliations:** 1 Department of Otorhinolaryngology, Head and Neck Surgery, Heidelberg University Hospital, Heidelberg University, 69120 Heidelberg, Germany; Chao.rong@med.uni-heidelberg.de (C.R.); marief4@web.de (M.M.); Gerhard.dyckhoff@med.uni-heidelberg.de (G.D.); cem.bulut@slk-kliniken.de (O.C.B.); Peter.plinkert@med.uni-heidelberg.de (P.P.); Jochen.hess@med.uni-heidelberg.de (J.H.); 2 Institute of Pathology, Heidelberg University Hospital, Heidelberg University, 69120 Heidelberg, Germany; Christa.flechtenmacher@med.uni-heidelberg.de; 3 Molecular Diagnostics of Oncogenic Infections, German Cancer Research Center (DKFZ), 69120 Heidelberg, Germany; D.Holzinger@dkfz-heidelberg.de; 4 Department of Otorhinolaryngology, SLK Kliniken, Am Gesundbrunnen, 74078 Heilbronn, Germany; 5 Department of Oral and Cranio-Maxillofacial Surgery, Heidelberg University Hospital, Heidelberg University, 69120 Heidelberg, Germany; Dominik.horn@med.uni-heidelberg.de; 6 Molecular Mechanisms of Head and Neck Tumors, German Cancer Research Center (DKFZ), 69120 Heidelberg, Germany

**Keywords:** oropharyngeal squamous cell carcinoma, MAPK/ERK, AKT, prognostic biomarker, human papillomavirus, tissue microarray

## Abstract

Human papillomavirus (HPV)-related oropharyngeal squamous cell carcinoma (OPSCC) forms a distinct tumor entity with better survival clinical outcome. Numerous underlying molecular mechanisms have been postulated for differences in treatment response, but the impact of MEK/ERK signaling, a main driver of carcinogenesis in various cancers including OPSCC and key player mediating therapy resistance remains elusive. In a retrospective experimental cohort study, primary tumor samples from OPSCC patients (*n* = 124) were available on tissue microarrays (TMAs) and expression levels of phosphorylated ERK1/2 (pERK1/2) were detected by immunohistochemical staining. Correlations of pERK1/2 expression patterns with clinicopathological features and clinical outcome were evaluated by statistical analysis. A low pERK1/2 expression was strongly associated with HPV-related OPSCC, while primary tumors with high pERK1/2 staining showed a distinctly worse survival outcome and were associated with higher cellular differentiation. Co-activation of both ERK1/2 and AKT was a common event and was associated with unfavorable prognosis in our cohort. However, the combinatorial analysis of pAKT (Ser473) and pERK1/2 did not strengthen the predictive power of pERK1/2, suggesting that pERK1/2 plays a more significant function in OPSCC. In summary, our data provide a compelling experimental and statistical evidence that low levels of tumor cell intrinsic ERK1/2 activation contribute at least in part to the favorable outcome of HPV-related OPSCC. On the other hand, presented findings indicate that non-HPV-related OPSCC with elevated ERK phosphorylation are at high risk for treatment failure and might benefit from targeted therapy of MEK/ERK signaling.

## 1. Introduction

Head and neck squamous cell carcinomas (HNSCC) are among the most common solid cancers worldwide with an annual incidence of over 600,000 cases [1]. Besides the two well-established risk factors, tobacco and alcohol consumption, which account for more than 70% of all HNSCC [2], infection by high-risk types of human papillomavirus (HPV), in particular, HPV16, is a causative factor for an increasing incidence of oropharyngeal squamous cell carcinoma (OPSCC) [3,4]. Recent studies showed HPV-related OPSCC to be a distinct clinical and molecular entity [5,6]. Patients affected by HPV-related OPSCC tend to be younger and have better outcomes. This has led to the introduction of de-escalation strategies to reduce long-term toxicity [7,8,9]. However, the global 5-year-survival rate for all HNSCC sites is only 40–50% [10], although there are substantial differences among countries. Global survival rates are comparable with the survival in Germany where our study was based [11]. Current therapeutic paradigms do not adequately address the distinct clinical and biological heterogeneity of HNSCC with varying treatment responses [12,13]. Diagnosis of most HNSCC at more advanced tumor stages also contributes to the unfavorable prognosis. Since therapeutic resistance frequently develops, the successful treatment of HNSCC is significantly impaired [14]. Therefore, there is an urgent demand in valuable biomarkers and therapeutic targets for HNSCC patients.

Mitogen-activated protein kinase (MAPK) cascade is a critical pathway for transduction extracellular signals to cellular responses, which plays a significant role in tumor cell survival, proliferation, and resistance to current therapies [15]. Amongst those are the MEK/ERK1/2 signaling components. They are the final effectors of the MAPK signaling pathway. Previous studies addressing radioresistance in HNSCC provided compelling experimental evidence that activation of MEK-ERK1/2 signaling mediates the inadequate response to therapy [16]. Moreover, inhibition of irradiation-induced MEK-ERK1/2 activation, significantly suppressed colony forming ability, and enhanced apoptosis in epithelial cancer cells [17,18,19]. Activation of the phosphatidylinositol-3-kinase (PI3-K)/protein kinase B (AKT) pathway plays a central role in numerous cellular processes, including metabolism, cell growth, apoptosis, survival, and differentiation [20]. The cascade is also known for impacting on radioresistance development in HNSCC [21,22,23]. HPV-positive oropharyngeal SCC showed activating mutations of PIK3CA in 56% [24]. In a recent study, we found pAKT (Ser473) expression serves as an independent prognostic marker for progression-free survival in OPSCC [25]. So far, only inhibitors of the epidermal growth factor receptor (EGFR) upstream of MAPK/ERK signaling have gained approval for HNSCC treatment [26]. Reliable molecular biomarkers for early detection as well as for the response to treatment are lacking for HNSCC. Activation of MAPK/ERK signaling is detrimental to response characteristics in HNSCC and MEK/ERK inhibitors are actually present in clinical trials. Hence, it is obvious to examine the impact of activated MAPK/ERK as a prognostic biomarker. To the best of our knowledge, there are no data yet available on how activation of ERK1/2 by phosphorylation is associated with clinical parameters and particularly with the HPV status in OPSCC. In the present study, we aim to shed new light on the role of ERK1/2 activation in relation to the HPV status as a potential predictive biomarker.

## 2. Results

### 2.1. ERK1/2 Phosphorylation in Primary Tumors of Oropharyngeal Squamous Cell Carcinoma (OPSCC) Patients Is Associated with Human Papillomavirus (HPV) Status and Histopathological Grading

**Figure 1:**
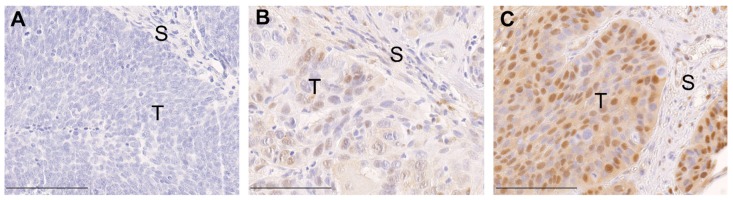
Immunohistochemical staining of pERK1/2 on tissue microarrays. Representative pictures of primary tumor sections with negative pERK1/2 immunostaining (**A**) and moderate pERK1/2 immunostaining (**B**); strong pERK1/2 immunostaining (**C**) (brown signal represents pERK1/2 protein expression; T: tumor; S: stroma; bars indicate 100 μm).

Immunohistochemical analysis of TMAs revealed a heterogeneous staining pattern of phosphorylated ERK1/2 (pERK1/2), ranging from undetectable to prominent staining intensity (Figure 1). Evaluation of pERK1/2 expression according to the relative number of positive tumor cells and the staining intensity was determined by a final immunoreactivity score (IRS) for OPSCC patients. The patients were divided into two subgroups depending on different IRS with pERK1/2 ^high^ (*n* = 68) and pERK1/2 ^low^ (*n* = 56) for further analysis. Chi-square analysis showed pERK1/2 expression is significantly associated with HPV status (*p* < 0.001), and histopathological grading (*p* = 0.039). Our findings indicate that a high pERK1/2 IRS was enriched in non-HPV-related OPSCC as compared to their HPV-related counterparts and was linked with good differentiation (Table 1).

### 2.2. ERK1/2 Phosphorylation Is Associated with Unfavorable Clinical Outcome

**Figure 2:**
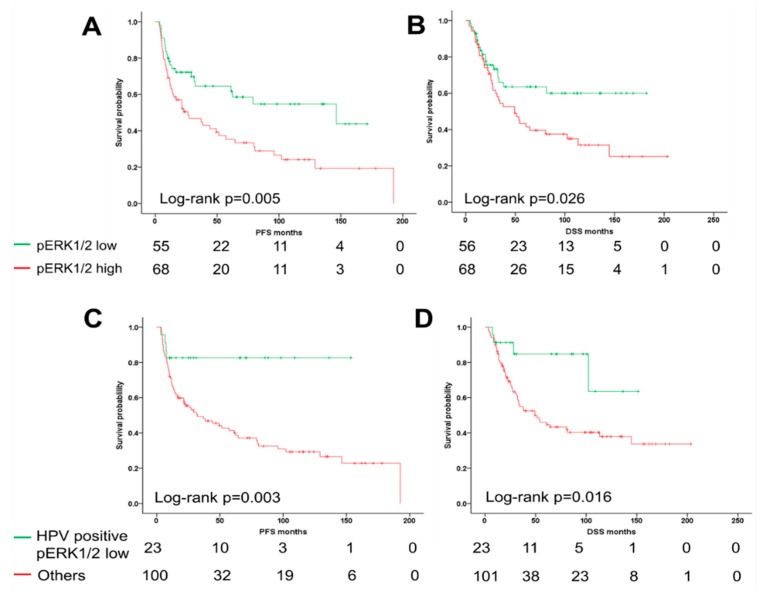
Correlation between pERK1/2 expression and survival of oropharyngeal squamous cell carcinoma (OPSCC) patients. The differences in progression-free (**A**) and disease-specific survival (**B**) between pERK1/2 staining patterns were plotted by a univariate Kaplan–Meier analysis and log-rank test. Human papillomavirus (HPV)-related OPSCC with low pERK1/2 expression show more favorable progression-free (**C**) and disease-specific survival (**D**). The number of survivors after a certain time interval (given in months) is displayed.

To address the prognostic value of pERK1/2 expression in OPSCC, survival analyses for subgroups with pERK1/2 ^low^ or pERK1/2 ^high^ were conducted by Kaplan–Meier plots and log-rank testing. In line with the close relationship in HPV-related OPSCC, a pERK1/2 ^low^ staining pattern correlated significantly with more favorable progression-free survival (PFS) (*p* = 0.005) and disease-specific survival (DSS) (*p* = 0.026) as compared to pERK1/2 ^high^ staining pattern (Figure 2A,B). Interestingly, up to 71% of pERK1/2 ^low^ staining pattern cases are HPV-positive OPSCC. The subgroup of pERK1/2 ^low^ and HPV-positive patients were compared with another subgroup by Kaplan–Meier plots and log-rank test. A combinatorial subgroup analysis revealed that the subgroup of pERK1/2 ^low^ and HPV-positive patients have distinctly favorable clinical prognosis (Figure 2C,D).

### 2.3. The ERK1/2 Phosphorylation Is a Prognostic Biomarker for the Survival of Oropharyngeal Squamous Cell Carcinoma (OPSCC) Patients Dependent on Human Papillomavirus (HPV) Status

**Figure 3:**
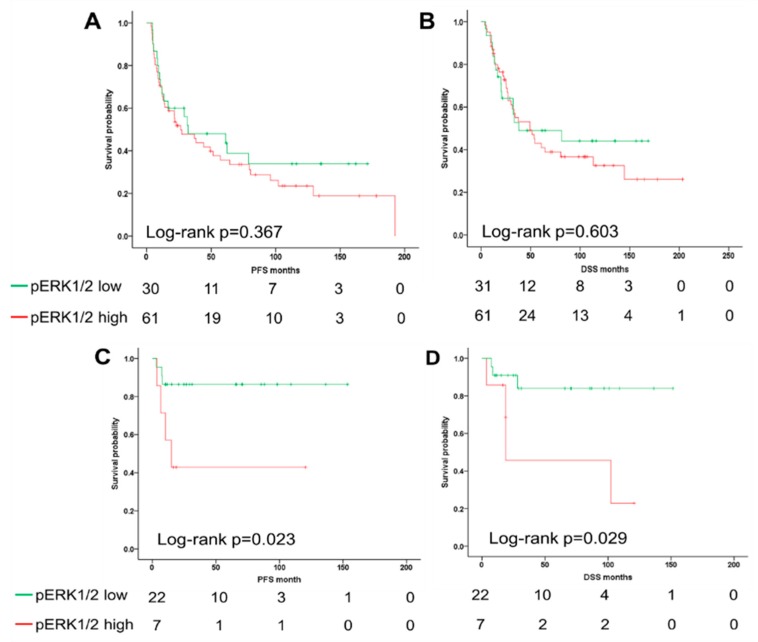
Correlation between pERK1/2 expression and survival of human papillomavirus (HPV)-negative and positive oropharyngeal squamous cell carcinoma (OPSCC) patients. Kaplan–Meier plots show the difference in disease-specific (DSS) and progression-free survival (PFS) between subgroups low and high pERK expression in HPV-negative (**A**,**B**) and positive (**C**,**D**) OPSCC patients. The number of survivors after a certain time interval (given in months) is displayed.

Consequently, univariate analyses revealed a significant correlation between higher T status, current smoking, non-HPV-related OPSCC, and a pERK1/2 ^high^ staining pattern with shorter PFS and DSS, respectively, while lymph node metastasis was significantly associated with DSS (Table 2). In order to adjust for all significant clinical parameters, a multivariate Cox regression model was fitted (Table 3). Only smoking status was found to be an independent risk factor for both PFS (hazard ratio (HR) = 2.684; 95% confidence intervals (CI) = 1.350–5.334; *p* = 0.005), and DSS (HR = 2.589; 95% CI = 1.213–5.529; *p* = 0.014). A higher T stage and a positive N status serve as an independent risk factor for reduced PFS (HR = 1.751; 95% CI = 1.028–2.984; *p* = 0.039) or DSS (HR = 2.637; 95% CI = 1.248–5.570; *p* = 0.011), respectively. Multiple studies have demonstrated that HPV infection serves as an independent prognostic biomarker for OPSCC patients [6,27,28]. However, HPV status was not an independent predictor in the multivariate analysis from our cohort, which might be due to the close correlation with pERK1/2 IRS. To address the question, whether pERK1/2 expression serves as an unfavorable factor for the clinical outcome of OPSCC patients depending on HPV status, we performed Kaplan–Meier analysis for PFS and DSS in the subgroup of OPSCC patients with or without HPV infection. Kaplan–Meier survival analysis for HPV-negative OPSCC showed that pERK1/2 expression is not associated with PFS and DSS. However, high pERK1/2 expression correlated with poor PFS and DSS in HPV-positive OPSCC (Figure 3). These data suggested pERK1/2 being an indicator of poor survival dependent on the OPSCC’s HPV status.

### 2.4. Combinatorial Analysis of ERK1/2 and AKT Phosphorylation in Oropharyngeal Squamous Cell Carcinoma (OPSCC)

**Figure 4:**
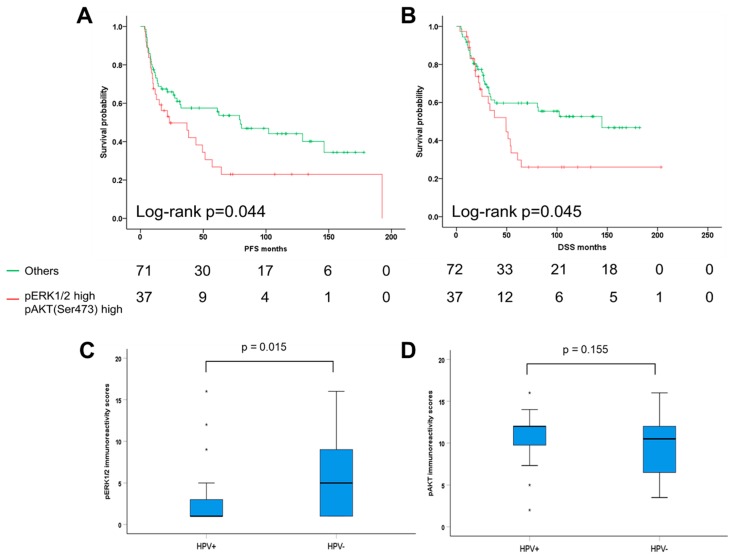
Correlation survival analysis between ERK1/2 and AKT phosphorylation. Kaplan–Meier plots for progression-free (**A**) and disease-specific survival (**B**) revealed that patients with co-activation of both pathways MEK1/ERK1/2 and PI3K/AKT have an unfavorable clinical outcome as compare to others. The number of survivors after a certain time interval (given in months) is displayed. Boxplot depicts the immunoreactivity scores of pERK1/2 (**C**) and pAKT (Ser473) (**D**) between human papillomavirus (HPV) negative and positive oropharyngeal squamous cell carcinoma (OPSCC) patients (HPV+: HPV-positive, HPV−: HPV-negative).

PI3k/AKT pathway is one of the major signaling cascades overactivated in various tumor entities including OPSCC [29]. To gain insight into the correlation of PI3k/AKT and MEK1-ERK1/2 pathway in OPSCC, pERK1/2 staining patterns were compared with data for pAKT (Ser473) phosphorylation, which were assessed previously [25]. IRS data for both pERK1/2 and pAKT (Ser473) were available for 109 cases of our OPSCC cohort. Although correlation analysis did not reveal any statistical significance association (Pearson correlation *p* = 0.590; Spearman’s correlation *p* = 0.608), distinct subgroups with either pERK1/2 ^high^ pAKT (Ser473) ^high^ (*n* = 37), pERK1/2 ^high^ pAKT (Ser473) ^low^ (*n* = 22). pERK1/2 ^low^ pAKT (Ser473) ^high^ (*n* = 28), and pERK1/2 ^low^ pAKT (Ser473) ^low^ (*n* = 22) were identified. OPSCC with a pERK1/2 ^high^ pAKT (Ser473) ^high^ staining pattern had a significantly worse PFS and DSS as compared to all other staining patterns (Figure 4A,B). However, compared to tumors who solely express high pERK levels, co-activation of both pathways did not show worse survival outcome and was not significantly correlated with any clinical and histopathological features (Tables S1 and S2). In a multivariate Cox regression model, pERK1/2 expression was a prognostic factor for PFS and DSS independent of pAKT (Ser473), while coactivation of both pathways was not an independent risk factor for the unfavorable clinical outcome of OPSCC patients (Tables S3 and S4). 

Concerning the expression of pERK1/2 and pAKT (Ser473) in subgroups of HPV negative or positive patients, we found a significantly higher IRS of pERK1/2 in HPV negative tumors as compared to that in HPV positive tumors (Figure 4C). There is no significant difference of pAKT (Ser473) expression between HPV negative and positive OPSCC (Figure 4D). In detail, we found 38% (*n* = 31) of HPV-negative tumors (*n* = 82) showing high expression levels for both pERK1/2 and pAKT (Ser473). 54% (*n* = 13) of HPV-related samples (*n* = 24) displayed high levels of pAKT (Ser473) while pERK1/2 expression was low (Table S5). Correlation analyses revealed that there was no significant correlation between expression of pERK1/2 and pAKT (Ser473) in HPV-negative OPSCC (Pearson correlation *p* = 0.432; Spearman’s correlation *p* = 0.331) and in HPV-positive tumors (Pearson correlation *p* = 0.657; Spearman’s correlation *p* = 0.985), respectively. In summary, our data indicate that PI3k/AKT and MEK1-ERK1/2 pathways are relatively independent signaling cascades in OPSCC, while pERK1/2 expression is more significantly associated with survival of OPSCC patients. In addition, we made use of the publicly available genomic database from The Cancer Genome Atlas (TCGA) and analyzed the genomic dataset from TCGA-HNC provided by the cBio Cancer Genomics Portal (http://cbioportal.org) [24,30,31]. In 37% of cases, genetic alterations (copy number variation and single-nucleotide variant) were found for PIK3CA, in 4% and 2.5% of cases genetic alteration were detected for AKT1 and AKT2, respectively (Figure S1A). However, no significant association was observed between PIK3CA /AKT1/AKT2 genetic alterations and protein levels (Figure S1B–G). No significant difference in overall survival was found in the two subgroups with and without alterations in PIK3CA gene and AKT1&2 genes (Figure S1H).

## 3. Discussion

In the present study, we found that HPV-related OPSCCs express lower pERK1/2 staining patterns than non-HPV-related counterparts, which suggested that well-documented differences in the survival outcome might be in part attributed to lower activation of MAPK/ERK signaling as a cancer cell intrinsic feature. MAPK/ERK signaling is critical for survival, dissemination, and resistance to therapy in numerous human cancer cells, and ERK1/2 phosphorylation results in the activation of multiple downstream substrates that are critically implicated in these processes [32]. Increased pERK1/2 and total ERK1/2 expression have been associated with radioresistance and poor prognosis in nasopharyngeal carcinoma [33] and clival chordomas [34]. This is in line with our previous results demonstrating that ERK1/2 activation impairs radiosensitivity of HNSCC cells [18,35]. There is evidence for pERK1/2 being a prognostic biomarker in several malignancies, including breast cancer [36,37,38,39], malignant melanoma [40], and hepatocellular carcinoma [41]. High expression of pERK1/2 enhances tumorigenicity and metastasis and has been linked to poor prognosis in esophageal cancer [42], hepatocellular carcinoma [43], and ovarian cancer [44]. However, it is not known how ERK signaling is associated with survival outcome in HNSCC or OPSCC, especially in relation to their HPV status. 

Our study describes activated ERK1/2 as a strong independent risk factor for poor prognosis in OPSCC. In detail, 68 of the 124 OPSCC samples showed high pERK1/2 IRS which had a significantly reduced progression-free and overall survival. The observed HR of 2.042 (95% CI 1.229–3.392) reflects an increase in the hazard for death by factor 2.042 for each level of the pERK1/2 high scores in the PFS interval and HR of 1.844 (95% CI 1.068–3.185) in the DSS interval. Specifically, tumor size, smoking, HPV association, and high pERK1/2 expression were determined to be risk factors in our cohort, calculated by univariate Cox regression analysis for PFS and OS. Not surprisingly, we found nicotine consumption to serve as an independent risk factor for survival outcome of OPSCC patients. So far, HPV status has been demonstrated as the most suitable independent predictor of survival in OPSCC patients. HPV-positive OPSCC have a significant survival advantage over HPV-negative tumors, with a 58% reduction in mortality risk [45]. HPV-related HNSCC tumors have a higher response to treatment as ionizing radiation that might explain their favorable outcomes [46]. High pERK1/2 IRS were strongly associated with HPV-negative OPSCC (61 out of 68 samples, *p* < 0.001). Vice versa, in HPV-related samples we found low pERK1/2 expression in 22/29 cases. Proliferation of HPV-positive cancer cells is mainly triggered by viral oncoproteins, in particular E7 [47]. As a consequence, activation of signaling cascades, including MAPK/ERK driving cell cycle progression seems to be less relevant in HPV-positive OPSCC which is supported by the fact that there are less genetic alterations in regulators of MAPK signaling, e.g., EGFR [48]. EGFR alterations (high gene copy numbers, overexpression) have been even found to be inversely correlated to HPV status in OPSCC [7]. Furthermore, ERK phosphorylation has been shown to be impaired by viral oncoproteins. The binding partner of E6, the ubiqutin ligase UBE3A was reported to dampen down basal level ERK activation through removing the p53 tumor suppressor protein in cervical cancer cells [49]. Our assumption concerning the relationship between ERK activation and HPV is supported by our finding regarding pERK1/2 expression and histopathological grading. High pERK1/2 levels were significantly associated with better cellular differentiation (G1/2) while poorly differentiated tumors were linked to low pERK1/2 expression. It has been shown that HPV-driven HNSCC frequently have a poorly differentiated histopathology [50,51] which is in line with our data.

The EGFR as upstream of ERK1/2 and AKT is expressed in more than 90% of all HNSCC [52,53]. Few data exist on the relationship between EGFR and HPV-induced oropharyngeal cancers. Several studies indicate that there is an inverse correlation between HPV infection and EGFR protein expression [54,55]. The relationship of HPV status with EGFR protein expression and survival outcome has been addressed in a few studies. These suggest that the best outcomes are observed in patients with HPV-related OPSCC with low EGFR expression and the worst in HPV-negative with high EGFR expression or high EGFR gene copy number, respectively [56]. This is in accordance with our findings. We assume that in our cohort MEK-ERK1/2 and PI3K/AKT are most likely activated by upstream factors such as EGFR in HPV-negative OPSCC showing worse survival outcome. Co-activation of both pathways MEK-ERK1/2 and PI3K/AKT was common in HPV-negative OPSCC, however, additional AKT (Ser473) phosphorylation did not increase the predictive power of pERK1/2. As OPSCC with co-activation of both pathways MEK-ERK1/2 and PI3K/AKT did not show worse outcome and were not significantly correlated with clinical and histopathological features we propose that these patients might not benefit from targeted therapy against MAPK/ERK. In summary, we demonstrated that phosphorylation of ERK1/2 is a rare event in HPV-positive OPSCC but quite frequent in HPV-negative tumors. Our data support the hypothesis that low levels of intrinsic ERK1/2 activation contribute partially to the positive prognosis of HPV-associated OPSCC. 

Our study was limited by the small sample size and the fact that not all of the patients received intensity-modulated radiation therapy causing better tumor targeting. However, we were able to identify activated ERK1/2 as a strong prognostic indicator in OPSCC for the first time. As we already suggested from previous in vitro and ex vivo data, the utilization of small molecule inhibitors against MEK-ERK1/2 kinases might prove itself as a promising specific therapeutic agent. The subgroup of patients which is supposed to benefit most is HPV-negative and show high pERK1/2 expression according to our findings. We assume that activated ERK1/2 decreases radiosensitivity, especially in HPV-negative OPSCC. OPSCC patients with HPV-negative but high pERK expression may be candidates for MEK-targeted therapy because such tumors show a more aggressive clinical course and are likely to respond to ERK inhibition. Further studies in a larger cohort of clinical samples are necessary to address the clinical significance of varying degrees of ERK1/2 pathway activation.

## 4. Materials and Methods

### 4.1. Patient Samples

Patients with primary OPSCC who were diagnosed and treated between 1990 and 2008 were comprised in the retrospective study cohort. Samples were obtained at the Department of Otorhinolaryngology, Head and Neck Surgery of Heidelberg University Hospital during diagnostic or therapeutic procedures. Biopsies of non-surgically treated patients, as well as samples of patients who underwent tumor surgery, were selected consecutively. All subjects gave written informed consent for data collection as it is a standard procedure in our department. Patients with suspicious clinical findings who underwent diagnostic endoscopy and/or patients before tumor surgery with a histologically confirmed diagnosis of OPSCC were asked to consent. The protocol was approved by the Ethics Committee of the Medical Faculty of the University of Heidelberg (Ethic vote: 176/2002) in accordance with the declaration of Helsinki. Experimental treatment procedures were not part of this study. The patients were treated according to the guidelines for head and neck cancer. The final analysis was based on 124 patients with OPSCC (Table S6). Clinical and therapeutic follow-up of the cohort was assessed retrospectively. 83 patients received surgery with/without adjuvant radio(chemo)therapy, and in 41 patients with poor performance status or with nonresectable tumors, definitive radiation and/or chemotherapy were applied. 3 patients received neoadjuvant treatment (i.e., induction chemotherapy/interleukin therapy). The cohort did not include recurrences or specimens from salvage surgery. 

### 4.2. Human Papillomavirus (HPV) Genotyping and HPV16 RNA Analysis

The HPV status for the cohort was assessed and reported previously [27]. In short DNA and RNA were isolated using QIAamp DNA Mini Kit and RNeasy Mini Kit (Qiagen, Venlo, The Netherlands). BSGP51/61-PCR/Multiplex HPV genotyping was used for determination of HPV DNA status, including the amplification of 54 mucosal HPV types and the b-globin gene as a control for the quality and quantity of genomic DNA [25]. For the assessment of HPV RNA status, HPV16 E6*I mRNA transcripts were determined by a nucleic acid sequence-based amplification assay, as described recently [27]. Viral DNA- and transcript-positive samples were defined as HPV-positive, viral DNA-negative or DNA-positive, but transcript-negative samples were defined as HPV-negative, according to Holzinger et al., 2012 [27].

### 4.3. Tissue Microarray and Immunohistochemistry

Tissue microarrays (TMAs) were prepared as described previously [57,58]. TMAs were stained with an anti-phospho-p44/42 MAPK (ERK1/2) (Thr202/Tyr204) antibody (Cell Signaling, Cambridge, UK, #9101) and immunostaining was visualized with the TSA Amplification Kit (Perkin Elmer, Rodgau, Germany) and DAB peroxidase substrate (Vector Laboratories, Burlingame, CA, USA) according to the manufacturer’s instructions. Counterstaining was done by hematoxylin to visualize tissue integrity. Stained TMAs were scanned using the Nanozoomer HT Scan System (Hamamatsu Photonics, Japan) and were evaluated by three independent observers using the NDP Viewer software (version 1.1.27, Hamamatsu Photonics, Japan). Evaluation considered the relative amount of positive cancer cells (score 1 = no positive cell, score 2 ≤ 33%, 33% > score 3 ≤ 66%, score 4 > 66%) and the staining intensity (score 1 = no, score 2 = low, score 3 = moderate, score 4 = high) [59]. Both values were multiplied to calculate the final immunoreactivity score (IRS, range 1–16), and the cut-off value for further analysis was pERK ^high^ > 3 and pERK ^low^ ≤ 3. To take the heterogeneity of tumors into account, three TMA slides including at least three spots for each OPSCC were analyzed. Patients whose three IRS had a large variation were excluded from further analysis. Data on the IRS for pAKT (Ser473) were available from a previous study [25,60]. 

### 4.4. TCGA-HNSCC Dataset Analysis

The publically available TCGA-HNSCC dataset was analyzed via the cBio Cancer Genomics Portal (http://cbioportal.org). Analyses and visualizations were performed according to the guidelines and protocols [30,31]. Additional information concerning the level of the data and methods used in the process can be found at TCGA website (https://tcga-data.nci.nih.gov/tcga/).

### 4.5. Statistical Analysis

SPSS 22 (IBM SPSS Statistics for Windows, Version 22.0. Armonk, NY, USA: IBM Corp.) was used for statistical analysis. Correlations between phospho-ERK expression and clinical and histopathological parameters (gender, age, tumor size, lymph node metastases, tumor grade) as well as risk factors (smoking, alcohol consumption, HPV status) were calculated by cross tables and chi-square test. *p*-values < 0.05 were considered significant. Disease-specific survival (DSS) was calculated as the time from date of primary OPSCC diagnosis to the date of tumor-related death within the follow-up interval (events). Survival times of patients who were alive or were dead due to tumor-unrelated reasons were censored. Progression-free survival (PFS) was calculated from the date of primary OPSCC diagnosis to the date of lymph node or distant metastasis, the first local recurrence, second primary tumor or data of OPSCC-related death within follow-up period (events), to the date of tumor-unrelated death or without progression were censored. DSS and PFS data were plotted by Kaplan–Meier survival curves. Differences between groups were assumed using log-rank testing. Univariate and multivariate Cox proportional hazard models were applied to define the interdependence between multiple parameters and prognosis by using the approach “enter”. 

## 5. Conclusions

In summary, our data demonstrate for the first time that low level of tumor cell intrinsic ERK1/2 activation contributes at least in part to the favorable outcome of HPV-related OPSCC. On the other hand, presented findings provide a proof-of-concept that non-HPV-related OPSCC with elevated ERK phosphorylation are at high risk for treatment failure and might benefit from targeted therapy of MEK/ERK signaling. However, further studies in a larger cohort of clinical samples are urgently needed to address the clinical significance of varying degrees of ERK1/2 pathway activation. It also will be worth establishing the promising preclinical models to gain more insight into the concept, whether OPSCC patients with HPV-negative but high pERK expression might benefit from MEK/ERK targeted therapy.

## Figures and Tables

**Table 1:** Association between pERK1/2 expression and clinicopathological features in oropharyngeal squamous cell carcinoma (OPSCC) patients, treated between 1990 and 2008 in Heidelberg (*n* = 124).

Clinicopathological Features	pERK1/2 ^low^	pERK1/2 ^high^	*p*-Value
Age(years)			0.060
<58	21	37	
≥58	35	31	
Gender			0.677
Male	43	50	
Female	13	18	
TNM status			
T1/T2	28	31	0.680
T3/T4	28	36	
missing		1	
N0	10	19	0.172
N+	46	48	
missing		1	
Pathological Grade			**0.039**
G1/G2	27	37	
G3	28	17	
missing	1	14	
Tobacco			0.293
Never/former	17	15	
Current	39	53	
Alcohol			0.371
Never/former	11	18	
Current	45	50	
HPV			**<0.001**
HPV-	31	61	
HPV+	22	7	
missing	3		
Therapy			0.231
RT	51	57	
Non-RT	5	11	

TNM: Classification of Malignant Tumors, HPV: human papillomavirus, RT: Radiotherapy; Statistically significant values are represented in bold.

**Table 2:** Univariate Cox regression analysis of progression-free survival (PFS) and disease-specific survival (DSS) for oropharyngeal squamous cell carcinoma (OPSCC) patients (*n* = 124).

Factors	PFS		DSS	
	HR (95% CI)	*p*-Value	HR (95% CI)	*p*-Value
Age(years)	0.925 (0.576–1.484)	0.747	1.037 (0.621–1.732)	0.890
≥58 vs. <58				
Gender	1.494 (0.816–2.737)	0.193	1.797 (0.909–3.549)	0.092
male vs. female				
T status	2.000 (1.210–3.308)	**0.007**	2.321 (1.328–4.054)	**0.003**
T3–4 vs. T1–2				
N status	1.433 (0.793–2.588)	0.233	2.274 (1.115–4.636)	**0.024**
N+ vs. N0				
pathological Grade	0.842 (0.483–1.467)	0.543	0.928 (0.507–1.698)	0.808
G3 vs. G1–2				
Tobacco	3.094 (1.575–6.077)	**0.001**	2.781 (1.318–5.866)	**0.007**
Current vs. Never/former				
Alcohol	1.305 (0.712–2.392)	0.389	1.347 (0.699–2.594)	0.373
Current vs. Never/former				
HPV status	0.357 (0.163–0.782)	**0.010**	0.452 (0.205–0.996)	**0.049**
Driven vs. Non-driven				
Therapy	1.578 (0.722–3.451)	0.253	2.507 (0.908–6.922)	0.076
RT vs. Non-RT				
Phospho-ERK1/2	2.042 (1.229–3.392)	**0.006**	1.844 (1.068–3.185)	**0.028**
High vs. Low				

HR, hazard ratio; CI, confidence intervals; PFS: progression-free survival; DSS: disease-specific survival. Statistically significant values are represented in bold.

**Table 3:** Multivariate Cox regression analysis of progression-free survival (PFS) and disease-specific survival (DSS) for oropharyngeal squamous cell carcinoma (OPSCC) patients (*n* = 124).

Factors	PFS		DSS	
	HR (95% CI)	*p*-Value	HR (95% CI)	*p*-Value
T status	1.751 (1.028–2.984)	**0.039**	1.740 (0.969–3.122)	0.064
T3–4 vs. T1–2				
N status	1.656 (0.881–3.114)	0.117	2.637 (1.248–5.570)	**0.011**
N+ vs. N0				
Tobacco	2.684 (1.350–5.334)	**0.005**	2.589 (1.213–5.529)	**0.014**
Current vs. Never/former				
HPV status	0.535 (0.232–1.236)	0.143	0.615 (0.265–1.430)	0.259
Driven vs. Non-driven				
Phospho-ERK1/2	1.746 (1.009–3.023)	**0.046**	1.653 (0.923–2.958)	0.091
High vs. Low				

HR, hazard ratio; CI, confidence intervals; Statistically significant values are represented in bold.

## Supplementary Materials

The following are available online at https://www.mdpi.com/2072-6694/11/4/584/s1, Figure S1: Genomics analysis of PI3k/AKT in HNSCC by the cBio Cancer Genomics Portal. Table S1: Correlation analysis for pERK1/2 and pAKT (Ser473) expression and clinicopathological features (*n* = 109), Table S2: Univariate Cox regression analysis of progression-free and disease-specific survival for OPSCC patients (*n* = 109), Table S3: Multivariate Cox regression analysis of progression-free and disease-specific survival for OPSCC patients (*n* = 109), Table S4: Multivariate Cox regression analysis of progression-free and disease-specific survival for OPSCC patients (*n* = 109), Table S5: pERK1/2 and pAKT(Ser473) expression distribution in HPV negative and positive OPSCC patients, Table S6: Descriptive analysis of clinicopathological features of OPSCC cohort, treated between 1990 and 2008 in Heidelberg (*n* = 124).
