# Supplementary material for: Differential Activation of ERK Signaling in HPV-Related Oropharyngeal Squamous Cell Carcinoma
Source: Cancers (Basel). 2019 Apr 25;11(4):584. doi: 10.3390/cancers11040584 (PMC6520790; doi:10.3390/cancers11040584)
Supplement: Supplementary file 1 [file cancers-11-00584-s001.pdf]

# Supplementary Materials: Differential Activation of ERK Signaling in HPV-Related Oropharyngeal Squamous Cell Carcinoma

**Chao Rong, Marie Muller, Christa Flechtenmacher, Dana Holzinger, Gerhard Dyckhoff, Olcay Cem Bulut, Dominik Horn, Peter Plinkert, Jochen Hess and Annette Affolter**

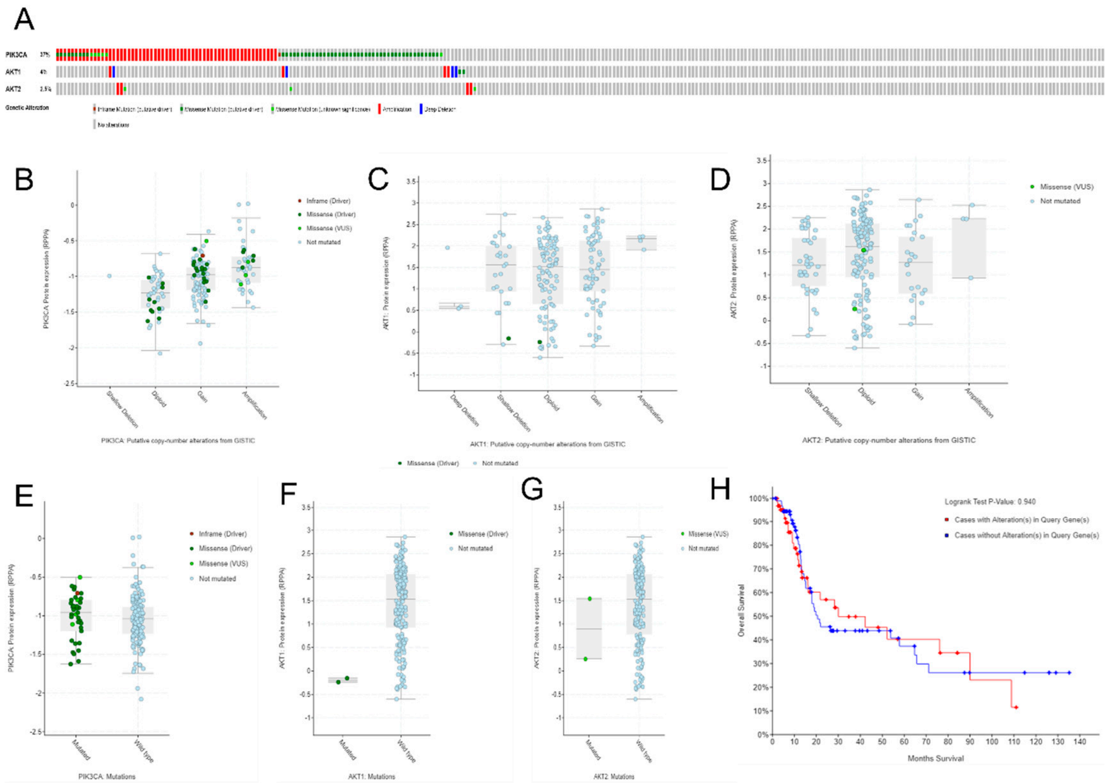


**Figure S1.** Genomics analysis of PI3k/AKT in HNSCC by the cBio Cancer Genomics Portal. (**A**) Oncomap indicates the frequency (%) of alterations for PIK3CA, AKT1 and AKT2 genes in the TCGA-HNC cohort ($n$ = 191), based on whole-exome sequencing data (http://www.cbioportal.org/). The boxplots show the association of protein levels and copy number variations as well as mutation status in PIK3CA (**B**,**E**), AKT1 (**C**,**F**) and AKT2 (**D**,**G**) genes. Differences in overall survival between subgroups with or without alterations in PIK3CA, AKT1&2 genes are plotted by Kaplan-Meier graph (**H**).

**Table S1.** analysis for pERK1/2 and pAKT (Ser473) expression and clinicopathological features ($n$ = 109).

| Clinicopathological Features | pERK1/2$^{high}$ pAKT$^{high}$ | Others | *p*-Value |
|---|---|---|---|
| Age(years) | | | |
| <58 | 19 | 32 | 0.494 |
| ≥58 | 18 | 40 | |
| Gender | | | |
| Male | 25 | 56 | 0.248 |
| Female | 12 | 16 | |
| TNM status | | | |
| T1/T2 | 17 | 32 | 0.881 |
| T3/T4 | 20 | 40 | |

| | | | |
|---|---|---|---|
| N0 | 7 | 16 | 0.689 |
| N+ | 30 | 56 | |
| Pathological Grade | | | 0.420 |
| G1/G2 | 19 | 38 | |
| G3 | 10 | 29 | |
| missing | 8 | 5 | |
| Tobacco | | | 0.486 |
| Never/former | 8 | 20 | |
| Current | 29 | 52 | |
| Alcohol | | | 0.789 |
| Never/former | 8 | 14 | |
| Current | 29 | 58 | |
| HPV | | | 0.247 |
| HPV- | 31 | 51 | |
| HPV+ | 6 | 18 | |
| missing | | 3 | |
| Therapy | | | 0.322 |
| RT | 31 | 65 | |
| Non-RT | 6 | 7 | |

RT: Radiotherapy.

**Table S2.** Cox regression analysis of progression-free and disease-specific survival for OPSCC patients (*n* = 109).

| **Factors** | **PFS** | | **DSS** | |
|---|---|---|---|---|
| | **HR (95% CI)** | ***p*-Value** | **HR (95% CI)** | ***p*-Value** |
| Age(years)<br>≥58 vs. <58 | 0.961(0.583–1.583) | 0.876 | 1.064(0.619–1.830) | 0.822 |
| Gender<br>male vs. female | 1.379(0.746–2.549) | 0.305 | 1.708(0.858–3.403) | 0.128 |
| T status<br>T3–4 vs. T1–2 | 1.881(1.116–3.171) | **0.018** | 2.293(1.272–4.132) | **0.006** |
| N status<br>N+ vs. N0 | 1.384(0.736–2.602) | 0.313 | 2.064(0.971–4.385) | 0.060 |
| pathological Grade<br>G3 vs. G1–2 | 0.742(0.413–1.334) | 0.319 | 0.750(0.393–1.433) | 0.384 |
| Tobacco<br>Current vs. Never/former | 3.359(1.596–7.072) | **0.001** | 2.861(1.290–6.341) | **0.010** |
| Alcohol<br>Current vs. Never/former | 1.071(0.558–2.056) | 0.837 | 1.090(0.548–2.171) | 0.806 |
| HPV status<br>Driven vs. Non–driven | 0.339(0.146–0.789) | **0.012** | 0.423(0.181–0.990) | **0.047** |
| Therapy<br>RT vs. Non-RT | 1.220(0.555–2.680) | 0.620 | 2.029(0.732–5.626) | 0.174 |
| p-AKT$^{high}$<br>High vs. Low | 1.117(0.673–1.856) | 0.668 | 1.220(0.700–2.124) | 0.483 |
| p-ERK1/2$^{high}$ p-AKT$^{high}$<br>vs. others | 1.686(1.009–2.817) | **0.046** | 1.745(1.006–3.028) | **0.048** |

HR, hazard ratio; CI, confidence intervals; Statistically significant values are represented in bold.

**Table S3.** Multivariate Cox regression analysis of progression-free and disease-specific survival for OPSCC patients ($n$ = 109).

| Factors | PFS | | DSS | |
|---|---|---|---|---|
| | **HR (95% CI)** | ***p*-Value** | **HR (95% CI)** | ***p*-Value** |
| T status<br>T3–4 vs. T1–2 | 1.748(1.028–2.973) | **0.039** | 2.114(1.160–3.852) | **0.014** |
| Tobacco<br>Current vs. Never/former | 2.725(1.276–5.819) | **0.010** | 2.371(1.055–5.327) | **0.037** |
| HPV status<br>Driven vs. Non-driven | 0.464(0.197–1.097) | 0.080 | 0.577(0.241–1.382) | 0.217 |
| p-ERK1/2$^{high}$ p-AKT$^{high}$<br>vs. others | 1.400(0.830–2.362) | 0.207 | 1.338(0.758–2.361) | 0.315 |

HR, hazard ratio; CI, confidence intervals; Statistically significant values are represented in bold.

**Table S4.** Multivariate Cox regression analysis of progression-free and disease-specific survival for OPSCC patients ($n$ = 109).

| Factors | PFS | | DSS | |
|---|---|---|---|---|
| | **HR (95% CI)** | ***p*-Value** | **HR (95% CI)** | ***p*-Value** |
| T status<br>T3–4 vs. T1–2 | 1.826(1.067–3.125) | **0.028** | 2.126(1.158–3.903) | **0.015** |
| Tobacco<br>Current vs. Never/former | 3.214(1.522–6.787) | **0.002** | 2.620(1.178–5.828) | **0.018** |
| p-AKT<br>High vs. Low | 1.064(0.630–1.798) | 0.816 | 1.098(0.621–1.942) | 0.748 |
| p-ERK1/2<br>High vs. Low | 2.067(1.204–3.550) | **0.008** | 1.855(1.038–3.315) | **0.037** |

HR, hazard ratio; CI, confidence intervals; Statistically significant values are represented in bold.

**Table S5.** pERK1/2 and pAKT(Ser473) expression distribution in HPV negative and positive OPSCC patients.

| Expression Distribution | HPV− | | HPV+ | |
|---|---|---|---|---|
| | **pERK1/2 $^{low}$** | **pERK1/2$^{high}$** | **pERK1/2 $^{low}$** | **pERK1/2$^{high}$** |
| pAKT (Ser473)$^{low}$ | 15 | 22 | 5 | 0 |
| pAKT (Ser473)$^{high}$ | 14 | 31 | 13 | 6 |

**Table S6.** Descriptive analysis of clinicopathological features of OPSCC cohort, treated between 1990 and 2008 in Heidelberg ($n$ = 124).

| Clinicopathological Feature | No of Patients (%) |
|---|---|
| Age(years) | |
| ≥58 | 58 (46.8) |
| <58 | 66 (53.2) |
| Sex | |
| Male | 93 (75.0) |
| Female | 31 (25.0) |
| Tobacco use | |
| Never/Former | 32 (25.8) |
| Current | 92 (74.2) |
| Alcohol use | |
| Never/Former | 29 (23.4) |
| Current | 95 (76.6) |
| Tumor size (T) | |
| T1-2 | 59 (47.6) |
| T3-4 | 64 (51.6) |

| | |
|---|---|
| missing | 1 (0.8) |
| Lymph node metastasis (N) | |
| N0 | 29 (23.4) |
| N+ | 94 (75.8) |
| missing | 1 (0.8) |
| HPV16 | |
| HPV-non driven | 92 (74.2) |
| HPV-driven | 29 (23.4) |
| n/a (missing) | 3 (2.4) |
| Localization | |
| Tonsils | 60 (48.4) |
| Tongue base | 21 (16.9) |
| Soft palate/ Uvula/palatoglossal fold | 23 (18.5) |
| Extending several anatomic regions | 19 (15.3) |
| Oropharynx NOS (not otherwise specified) | 1 (0.8) |
| Treatment | |
| No radiotherapy | 16 (12.9) |
| Radiotherapy | 108 (87.1) |

© 2019 by the authors. Licensee MDPI, Basel, Switzerland. This article is an open access article distributed under the terms and conditions of the Creative Commons Attribution (CC BY) license (http://creativecommons.org/licenses/by/4.0/).
